# Retraction: Association between Polymorphism of the Interleukin-13 Gene and Susceptibility to Hepatocellular Carcinoma in the Chinese Population

**DOI:** 10.1371/journal.pone.0181509

**Published:** 2017-07-12

**Authors:** Yan Deng, Ming Xie, Li Xie, Jian Wang, Taijie Li, Yu He, Ruolin Li, Shan Li, Xue Qin

After the publication of the article, the authors notified the *PLOS ONE* Editors that an error in the data analyses had been detected following a reanalysis of the data. While there are no concerns about the data themselves, the experimental and control groups were inadvertently switched during the original analysis. This error unfortunately lead to the opposite results being reported. As the analysis error impacts the overall conclusions of the study as published, the *PLOS ONE* Editors and an Academic Editor concluded that this article should be retracted from the literature.

In light of these issues, the authors and *PLOS ONE* Editors retract this article.
